# The Autonomic Nervous System Pulls the Strings to Coordinate Circadian HSC Functions

**DOI:** 10.3389/fimmu.2020.00956

**Published:** 2020-05-20

**Authors:** Andrés García-García, Simón Méndez-Ferrer

**Affiliations:** ^1^Tissue Engineering, Department of Biomedicine, University Hospital Basel, University of Basel, Basel, Switzerland; ^2^Wellcome Trust-Medical Research Council Cambridge Stem Cell Institute, Cambridge, United Kingdom; ^3^National Health Service Blood and Transplant, Cambridge, United Kingdom; ^4^Department of Haematology, University of Cambridge, Cambridge, United Kingdom

**Keywords:** autonomic nervous system, circadian, hematopoietic (stem) cells, adrenergic, cholinergic

## Abstract

As for many other adult stem cells, the behavior of hematopoietic stem and progenitor cells (HSPCs) is subjected to circadian regulatory patterns. Multiple HSPC functions, such as proliferation, differentiation or trafficking exhibit time-dependent patterns that require a tight coordination to ensure daily blood cell production. The autonomic nervous system, together with circulating hormones, relay circadian signals from the central clock—the suprachiasmatic nucleus in the brain—to synchronize HSC niche physiology according to light/darkness cycles. Research over the last 20 years has revealed how specific neural signals modulate certain aspects of circadian HSC biology. However, only recently some studies have started to decipher the cellular and molecular mechanisms that orchestrate this complex regulation in a time-dependent fashion. Here we firstly review some of the recent key findings illustrating how different neural signals (catecholaminergic or cholinergic) regulate circadian HSC egress, homing, maintenance, proliferation, and differentiation. In particular, we highlight the critical role of different neurotransmitter receptors in the bone marrow microenvironment to channel these neural signals and regulate antagonistic processes according to circadian cues and organismal demands. Then, we discuss the potential biological meaning of HSC circadian regulation and its possible utility for clinical purposes. Finally, we offer our perspective on emerging concepts in HSC chronobiology.

## Introduction

Throughout evolution, most species have developed the capacity to adapt their behavior and physiology to the day-night oscillations derived from earth's 24 h rotation. In mammals, these so-called circadian functions are synchronized by light-darkness shifts, and influenced by other factors, such as alimentary habits. The circadian system has at least two different levels of complexity. Centrally, the suprachiasmatic nucleus, located in the hypothalamus, is the anatomical structure that orchestrates the organism's circadian activities. This master clock receives photic input from photosensitive neurons in the retina through the retino-hypothalamic tract and processes this information to synchronize the circadian rhythms in peripheral tissues. However, most cells display at least one intrinsic or internal clock that influences their proliferation, differentiation and migration properties. This cellular clock is periodically reset by neuroendocrine signals emanating from the suprachiasmatic nucleus, which resets or synchronizes the peripheral oscillators throughout the body (1).

Already in the late 70s it was suspected that circadian rhythms might decisively influence stem cell functions and thus control both homeostasis and regeneration of peripheral tissues (2). Hematopoietic stem cells (HSCs) stand out as one of the most studied stem cell types. HSC functions are subjected to circadian fluctuations to ensure blood cell replenishment in mice and humans (3, 4). Although several studies have reported the expression of circadian clock genes (such as Bmal1, Clock, Cry1, Cry2, Per1, Per2, Rev-erb alpha, and Rev-erb beta) in mouse or human HSCs (5, 6), these genes do not exhibit clear light/darkness oscillatory patterns in HSCs (7). Indeed, a two-clock timing model was proposed to explain these differences in clock genes regulation (8). This model distinguishes between an endogenous clock, which is dominant in the neural system and is adjusted daily by photic signals, and an exogenous clock, which is more relevant for hemato-immune cells and is synchronized by environmental factors and/or changes in the organismal metabolism. According to this model, circadian rhythms in blood cells would be mostly regulated by the exogenous clock and rely on other oscillatory metabolic parameters and external cues (light-independent). However, as explained with more detail in the following sections, extensive research over the last few decades has demonstrated that photic cues and neural signals indeed regulate important circadian HSC functions in the bone marrow (BM) microenvironment.

The sympathetic branch of the autonomic nervous system connects the master clock in the central nervous system with peripheral organs to relay circadian information. For instance, adrenergic signals regulate clock gene expression in mouse liver (9), mouse heart (10), mouse and human osteoblasts (11, 12) and mouse brown adipose tissue (13). Similarly, β2-adrenergic receptor appears to regulate clock gene expression in BM stromal cells (14). Whilst adrenergic activity contributes to synchronize the peripheral oscillator, peripheral circadian rhythmicity can be maintained through other unknown mechanisms in the absence of adrenergic signaling (15).

Sympathetic nerves enter the BM associated with arteries and arterioles, and once inside the BM these nerves sprout throughout the BM space. BM sympathetic fibers mainly release noradrenaline in the BM, although both circulating noradrenaline and adrenaline can also reach the BM cavity via the vascular network (16). In sharp contrast, there is scarce neuroanatomical evidence of parasympathetic innervation in the BM. Although an indirect parasympathetic regulation of sympathetic postganglionary terminals cannot be excluded, the lack of parasympathetic fibers in the BM probably explains the scarce evidence for cholinergic regulation in the BM. It is important to note that not all autonomic cholinergic fibers are parasympathetic. In fact, some sympathetic fibers innervating the sweat glands or the periosteum undergo a neurotransmitter switch during postnatal development and start releasing acetylcholine (17, 18). Until recently, the role of these so-called sympathetic cholinergic nerve fibers in the BM has remained elusive. We have recently found that bone-associated cholinergic fibers play a crucial role in day/night oscillations of circulating HSCs and leukocytes. Indeed, sympathetic cholinergic fibers cooperate with the previously known sympathetic noradrenergic fibers and with central parasympathetic signals to orchestrate the circadian migration of HSCs and leukocytes between the BM and the bloodstream (19).

In this article, we discuss recent findings clarifying the roles of (nor)adrenergic and cholinergic signals to regulate different circadian HSC functions in BM niches. The future directions and perceived challenges for the future development of this multisystemic research are outlined below.

## Neural Regulation of Circadian HSC Traffic

The HSC is a unique type of adult stem cell since it preserves a remarkable migratory capacity at adulthood (20). HSCs circulate between the BM and the bloodstream in response to chemotactic signals, among which the C-X-C motif chemokine 12 (CXCL12) stands out as the most important chemokine. Although the biological function of HSC traffic is not yet fully understood, it is now accepted that HSCs circulate following circadian oscillations controlled by neural signals in different species, including humans (21, 22).

In the mouse system, sympathetic postganglionic terminals convey the circadian information from the suprachiasmatic nucleus to the BM, where noradrenaline is locally released. The adrenergic signals activate β3-adrenergic receptors in BM nestin+ mesenchymal stem cells (MSCs) and modulate CXCL12 production. This regulation leads to daily fluctuations in BM CXCL12 levels that inversely correlate with HSC numbers in the bloodstream. At daytime (the resting period in mice), light-induced noradrenergic signals downregulate CXCL12 expression via Sp1 transcription factor and promote HSC egress to the bloodstream. At night, BM CXCL12 levels are restored and HSCs preferentially return to the BM (also referred to as “HSC homing”). Remarkably, although circadian signals reach the BM through sympathetic terminals, their origin is set in the clock genes of the suprachiasmatic nucleus since normal circadian fluctuations are entrained by light and disappear in Bmal1^−/−^ mice (21). Importantly, the expression of the CXCL12 receptor CXCR4 in HSPCs is also subjected to circadian oscillations synchronized with the ligand. In the human system, these circadian oscillations are also detected but inverted compared with mice, as expected from humans and mice respectively being diurnal and nocturnal species (22). Interestingly, mouse and human circulating leukocytes also exhibit opposite circadian oscillations when they coexist in humanized mice (23), suggesting that cell-autonomous mechanisms are involved in species-specific circadian regulation.

Different types of adrenergic receptors cooperate in the regulation of HSC traffic. Besides the described role for β3-adrenergic receptor, β2-adrenergic receptor appears to regulate clock gene expression in BM stromal cells and cooperates with β3-adrenergic receptor in HSPC mobilization induced by granulocyte colony-stimulating factor (G-CSF), the agent most commonly used in the clinics (14). Interestingly, the same β-adrenergic receptor signaling was proposed to govern circadian leukocyte recruitment to mouse tissues (including the BM) during the nocturnal (active) phase in mice through stimulation of vascular endothelial cell adhesion (24). Therefore, these studies underscore the role of sympathetic noradrenergic signals as master regulators of circadian migration of leukocytes and HSCs/progenitors, which overall follow similar patterns. Daily neutrophil clearance by macrophages could be one event synchronizing leukocyte and HSPC trafficking. Macrophages are key cells to maintain HSCs in the BM (25, 26). Using mouse models, Casanova-Acebes et al. showed that aged neutrophils migrate at the end of the resting period to the BM, where they are engulfed by macrophages. This leads to the activation of LXR nuclear receptors on macrophages, which reduces the capacity of MSCs to retain HSPCs in the BM (27).

Despite these important findings, some critical aspects of circadian hematopoietic stem and progenitor cell (HSPC) traffic have long remained unanswered. For instance, A) how do BM stromal cells integrate similar noradrenergic signals at different circadian times and yet trigger distinct migratory processes (egress vs. homing)? In contrast to BM stroma-restricted expression of β3-adrenergic receptor, β2-adrenergic receptor is ubiquitously expressed (28, 29). As mentioned before, β2-adrenergic receptor cooperates with β3-adrenergic receptor in G-CSF-induced HSPC mobilization (14). Interestingly, adrenaline and noradrenaline can bind to β2-and β3-adrenergic receptors but they do so with very different affinities (30), suggesting that adrenaline and noradrenaline could have different effects on various cell types depending on the repertoire of adrenergic receptors expressed by the target cells. B) In that regard, do sympathetic neurotransmitters elicit similar responses if they signal in different BM cell populations or even through different β-adrenergic receptors in the same cell? C) Is noradrenaline the only neurotransmitter controlling circadian HSPC traffic?

An additional candidate neurotransmitter regulating HSPC traffic was hypothesized to be acetylcholine. Although modest, cholinergic innervation arising from the cholinergic forebrain and brain stem nuclei has been found in the suprachiasmatic nucleus. These cholinergic neurons have been proposed to play a role in the formation of time memory (memory of a specific time of the day associated with a certain event) (31). Recently, it was revealed that these cholinergic signals promote G-CSF-induced HSC mobilization from the BM through a glucocorticoid-signaling relay (32). Circulating glucocorticoids, such as corticosterone, follow circadian oscillations in blood and control HSPC proliferation through Notch signaling (33) and T cell distribution and activity through IL-7 receptor and CXCR4 (34). However, the precise role of circulating catecholamines in the circadian regulation of BM HSC niches was not well-understood.

In the context of physiological human hematopoietic cell trafficking between the BM and peripheral blood, one group of leukocytes (granulocytes, macrophages, natural killer cells, extrathymic T cells, gamma delta T cells and CD8+ cells) peak in the bloodstream at daytime, whilst another group (CD4+ T cells and B cells) exhibit an increase at night. Interestingly, those leukocytes with peak numbers in circulation at daytime express a higher density of adrenergic receptors, whereas those peaking at night carry a higher proportion of cholinergic receptors (35). Together, these data suggest a potential role for both adrenergic and cholinergic systems in the regulation of circadian hematopoietic cell trafficking.

**Figure 1:**
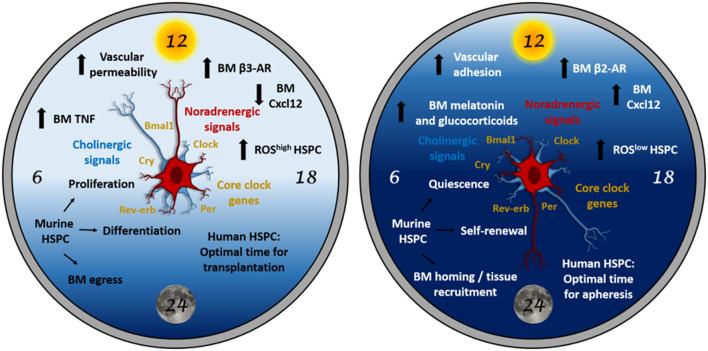
The “circadian clock” for HSPCs. HSPC biology is subjected to light-entrained circadian regulation orchestrated by the central clock (suprachiasmatic nucleus in the brain) and relayed to the BM by neural signals (noradrenergic and cholinergic), hormones and cytokines. At daytime (left panel), these signals trigger mouse HSPC proliferation, differentiation and egress to the bloodstream. At night (right panel), similar signals trigger different responses in the BM microenvironment resulting in mouse HSPC quiescence, self-renewal and homing to BM or peripheral tissues. In humans (diurnal), circadian oscillations occur in antiphase with mice (nocturnal). Therefore, the HSPC harvest yield from peripheral blood would be higher during the evening, whereas the homing efficiency of the transplanted HSPCs would be higher in the morning in humans. Although the possible role of clock genes in HSPCs remains under-investigated and might not be essential under homeostasis, clock genes (which regulate cell cycle) in HSCs might be important during stress or in hematological malignancies.

Indeed, we recently showed that the autonomic cholinergic nervous system (including parasympathetic nerves but also a proportion of sympathetic neurons releasing acetylcholine) cooperates with (nor)adrenergic sympathetic signals to orchestrate day/night oscillations of circulating HSPCs and leukocytes (19). Our findings reveal a dual cholinergic signaling that plays different roles in mice at day and night (Figure 1).

At night, inhibitory central cholinergic signals (parasympathetic) dampen sympathetic noradrenergic tone to reduce nocturnal egress of HSPCs and leukocytes from the BM into bloodstream. Whether central parasympathetic circadian activity is regulated by core clock genes in the suprachiasmatic nucleus or elsewhere remains unknown. Collectively, the results suggest that this cholinergic regulation cooperates with adrenergic signaling to drive HSPC and leukocytes recruitment to the BM during the active phase (night for rodents, day for humans). We propose that plasma adrenaline, which follows circadian fluctuations peaking at night (36, 37), preferentially binds to β2-adrenergic receptors to promote vascular adhesion and BM homing at night. Supporting this possibility, BM mRNA expression of β2- and β3-adrenergic receptors oscillate peaking at night or day, respectively, and genetic or pharmacological blockade of each receptor demonstrated their circadian time-dependent roles in HSC or leukocyte BM egress/homing (19).

In mice, light cues trigger sympathetic noradrenergic activity, which signals through β3-adrenergic receptor leading to predominant BM egress of HSPCs and leukocytes in the morning (21). However, light exposure seems to equally activate a subset of bone-associated sympathetic cholinergic neurons (18), whose functions had remained elusive. Our data show that light-triggered sympathetic cholinergic signals inhibit BM vascular cell adhesion and homing, which enable predominant egress of HSPCs and leukocytes into bloodstream at this circadian time (19).

Selective predominance of different β-adrenergic signaling at daytime and night, together with differential affinities of catecholamines for β-adrenergic receptors, may explain how sympathetic noradrenergic signals trigger BM egress or homing at different time points. Furthermore, we propose that a robust circadian rhythmicity in β-adrenergic receptor expression in BM stromal cells allows for these fine-tuned oscillations. Whereas, β3-adrenergic receptor expression is higher after light exposure (when BM egress becomes overriding), β2-adrenergic receptor expression predominates at night (when BM homing is maximum) (19). An additional role for sympathetic cholinergic innervation in this circadian regulation appears to be the inhibition of β3-adrenergic receptor expression at night.

## Neural Regulation of Circadian HSC Proliferation

Whilst it has been long appreciated that mouse and human HSPC proliferation (indirectly measured as DNA synthesis in the BM) follows a cyclic pattern, whether it is subjected to a bona-fide circadian rhythmicity has remained controversial for a long time (38–45). A potential role of the nervous system in this regulation was suggested after finding circadian fluctuations of catecholamines in human blood (46). Importantly, a pioneering study from Maestroni and colleagues had shown that noradrenergic stimulation promotes BM proliferation and protects against carboplatin-induced chemotherapy (47), which was later confirmed at the HSC level (48). Along these lines, noradrenaline concentration in the mouse BM correlates with the proportion of cycling hematopoietic cells (both peaking during the night or active phase for rodents), which altogether suggested that neural signals transduce the circadian information from the brain to the BM (16, 49). These observations also helped to understand the circadian-related differences in the capacity of murine HSPC to engraft in immunodeficient mice (50).

The Lapidot group confirmed these initial findings in humans using immature CD34+ cells (51). It is important to note that human and mouse hematopoietic cells appear to have a different clockwork, since inverted oscillations of circulating leukocytes have been described in immunodeficient mice carrying human and mouse leukocytes (52). Interspecies differences of stress-kinase regulation of reactive oxygen species (ROS), hypoxia-inducible factor 1α (HIF-1α) and clock gene–dependent regulation of the CXCL12 receptor CXCR4 appear to explain the opposite migratory patterns for human and mouse leukocytes (23). Noradrenaline and dopamine were found to increase human HSPC motility, proliferation, colony formation capacity and engraftment into immunodeficient mice (51). Interestingly, some neurotransmitters (such as noradrenaline) can exert these functions through directly activating β2-adrenergic receptors on hematopoietic cells (51). Therefore, the sympathetic nervous system regulates HSPC function through concerted actions on different receptors expressed by HSPCs (51) and their niche cells (19, 21).

The Lapidot laboratory has recently revealed the role of circadian BM noradrenergic signals in regulating the maintenance, proliferation and differentiation of mouse HSCs (53). Interestingly, two daily peaks of BM HSPC activity (one diurnal, one nocturnal) are preceded by transient increases of noradrenaline and tumor necrosis factor (TNF). Resembling circadian HSC traffic regulation through the BM niche (19, 21), the same signal appears to trigger different responses at each circadian time. For instance, TNF in the morning promotes HSPC proliferation, differentiation and migration, whilst TNF burst at night induces melatonin secretion to increase HSPC maintenance and self-renewal (53) (Figure 1). Overall, sympathetic noradrenergic signals cooperate with TNF levels to orchestrate blood cell replenishment during the day, whilst they serve to maintain the BM HSPC pool at night. The noradrenaline-TNF pathway promotes opposite HSC functions through a combination of mechanistic players including differential regulation of reactive oxygen species, vascular permeability, antigen expression and macrophage function. Based on the important contribution of the cholinergic neural system to day/night oscillations in HSPC and leukocyte migration (19), the possible regulation of HSPC proliferation and differentiation by cholinergic signals will be studied in the future.

## A Biological Meaning for Circadian HSC Regulation

The idiosyncrasy of the central master clock in mammals suggests that the biological benefit of these circadian oscillations might reside in its capacity to integrate and orchestrate different physiological functions that require precise coordination in order to adapt to organismal demands. The bone organ (including bone and BM) might actually represent a paradigmatic example of integrated physiology (54), since research over the past few decades has shown that bone and BM are coordinately regulated. For instance, whilst β3-adrenergic receptor was originally described to control circadian HSC traffic between the BM and the bloodstream, β2-adrenergic receptor has a prominent role in the circadian proliferation of bone-forming osteoblasts (11). Sequential proliferation and differentiation of MSC and osteolineage cells occurs at distinct circadian times. In rodents, preosteoblasts, the immediate proliferating precursors of osteoblasts, synthesize DNA primarily during the light cycle and divide during the subsequent dark cycle, whereas more immature osteoprogenitors display an inverted cycle (55). Perivascular nestin+ MSC are in contact with sympathetic fibers and are targeted by sympathetic efferent activity, whereas preosteoblasts are distributed closer to the bone surface (56). In addition, β3-adrenoceptor activation in nestin+ MSC inhibits their osteoblastic differentiation, while it does not affect their proliferation (56). In BM stromal cells, like osteoblasts, β2-adrenoceptor stimulation induces clock gene expression, whereas activation of β3-adrenoceptor downregulates *Cxcl12* (14). These data, together with the increased proliferation of nestin+ MSC after chemical sympathectomy (56), suggests that β2-adrenoceptor activation is responsible for circadian induction of nestin+ MSC proliferation, like previously reported for preosteoblasts (11). Therefore, it is possible that the differential localization, innervation and expression of β-adrenoceptors allow for circadian coordinated regulation of proliferation and differentiation of preosteoblasts and MSC, the last directly affecting HSC maintenance in the BM.

More broadly, many cytokines regulating bone accrual and bone formation work in a circadian fashion (with higher bone formation rate during the day in rodents) (57, 58). Therefore, since both systems are closely associated and even share multiple cell types and molecular pathways, it is reasonable to hypothesize that their circadian regulation is a control mechanism to ensure the coordinated and homeostatic function of the bone organ as a whole.

Alternatively, it has been proposed that daily HSC egress to circulation during the resting phase (light in rodents and night in humans) might contribute to regenerate BM stem cell niches (22, 59). Circulating HSCs would migrate to extramedullary tissues, where they could differentiate into mature hematopoietic cell types (60). It is important to note that the number of circulating HSCs at any time is very low, hampering the demonstration of their biological function. Furthermore, it is difficult to separate the impact of circadian rhythms *per se* from the physiological processes undergoing oscillations. For instance, sleep deprivation in mice reduces by 50% the capacity of HSCs to engraft into recipient mice undergoing normal day/night sleep cycles (61), but it is difficult to discern the effects of altered biological oscillations from the lack of regeneration due to sleep deprivation.

Another possible function of circadian HSPC traffic would be to maintain a pool of circulating progenitor cells to anticipate potential requirements derived from stress situations. In this sense, inflammatory monocytes have been found to follow circadian oscillations. However, this was shown to be mediated by intrinsic Bmal1 expression in myeloid cells, rather than light or environmental cues (62). In the aging context, some adult stem cells such as epidermal, muscle or liver stem cells do not lose their core circadian machinery, but their transcriptome switches from homeostasis genes to tissue-specific stress genes (e.g., DNA damage or autophagy) to adapt to age-related traits (63, 64). Supporting this idea, we found an adrenergic signaling switch in the aged BM niches from predominant β3- to β2-adrenergic receptor signaling, which facilitates myeloid cell expansion during aging (65).

As indicated above, clock genes in HSCs do not seem to play a crucial role in the homeostatic regulation of healthy HSCs but, as gatekeeper regulators of cell cycle, they can acquire a prominent role during stress (e.g., cancer, inflammation, etc.) (66). In contrast to healthy HSCs, the expansion of leukemic stem cells seems to be highly dependent on core clock genes. Bmal1 and Clock transcription factors were reportedly essential for leukemic stem cell self-renewal in acute myeloid leukemia (66). Therefore, anti-carcinogenic chronotherapies might take advantage of this susceptibility factor to target cancer stem cells without affecting normal stem cells. Furthermore, BM neuropathy is critical for the development of myeloproliferative neoplasms and acute myelogenous leukemia (67, 68), whilst altered noradrenergic signaling has been reported in aging (65, 69). Since neural signals are subjected to circadian fluctuations in the healthy/homeostatic BM, the disruption of neural circadian regulation might also contribute to disease/aging development, and thus represent a niche-related target for chronotherapies. In general, chronotherapy approaches in cancer have proven much more difficult than expected probably due to the interplay of different clocks and multiple entrainment signals in humans as a cause of notorious heterogeneity (52). It is important to note that the clock genes comprise only one of several cellular clocks. Other studies have found clock gene–independent mechanisms that maintain circadian cycles. For instance, ROS-regulating enzymes, such as peroxiredoxins, are ancient clocks that have been conserved throughout evolution (70). Therefore, future efforts in chronotherapy should take into account the interplay between the genetic clock and metabolic clocks, for instance.

## Potential Clinical Implications of Circadian HSC Rhythms

Since both circadian HSC proliferation and migration can impact HSC numbers in circulation, on a practical note it is recommended that clinicians consider harvesting blood (apheresis) later during the day to increase the stem cell yield in cases where HSC numbers might be limiting for the success of HSC transplantation procedures. Based on experimental evidence, a 2-3-fold higher HSPC number can be harvested by apheresis in the evening, compared with the morning apheresis, even after G-CSF-enforced HSC mobilization (22, 71). In contrast, it is expected that human HSC homing and subsequent engraftment in the BM would be more efficient early in the morning, when the BM transplantation procedure would be recommended for “poor engrafters” (24).

## Conclusions and Perspectives

In this article, we have summarized our current knowledge of the circadian regulation of HSPCs with a particular focus on the important yet intriguing role of sympathetic noradrenergic signals promoting HSPC proliferation, differentiation and egress during the resting phase (daytime for rodents), and HSPC maintenance, self-renewal and BM homing during the active phase (night for rodents) (Figure 1). The concerted regulation of HSPC function by noradrenergic signals requires fine-tuned control of adrenergic receptor expression, cytokines (e.g., TNF), hormones (like adrenaline or melatonin), ROS, vascular adhesion and permeability and, more broadly, BM HSC niche cells, such as MSCs, endothelial cells and macrophages. Moreover, the noradrenergic sympathetic system does act alone but cooperates daily with peripheral sympathetic cholinergic fibers and with central parasympathetic tone to orchestrate this complex HSPC regulation (19). A fascinating interplay among different organs takes place between brain, bone and BM to ensure that each organ's response meets the organismal demands.

The pathophysiological implications of the internal HSC clock remain largely unknown. In other systems, like the epidermis, the molecular clock machinery underlies the heterogeneity in the epidermal stem cell pool and could directly impact cell fate decisions, such as proliferation vs. dormancy. Particularly, differential clock gene expression leads to distinct responses of epidermal stem cells to transforming growth factor (TGF)-β and Wnt, which are signals that normally sustain their quiescence or proliferation, respectively (72). Furthermore, single-cell technology has revealed clock gene-dependent metabolic oscillations coordinated with DNA synthesis in these proliferating stem cells (73). It is possible that some of these regulatory mechanisms of epidermal stem cells are shared with HSCs, but this remains to be investigated. By the same token, the principles of neural regulation of HSC niches might be extrapolated in the future to other tissues, including the epidermis.

Whilst the influence of the neural system on HSPC differentiation has already been noted (53), the molecular drivers of cell commitment deserve further investigation. Likewise, cross-fertilization of ideas with other stem cell systems might pave the way for candidate molecular pathways involved. For instance, in both mouse and human embryonic stem cells, the *in vitro* pluripotent potential inversely correlates with the establishment of a circadian clock machinery, suggesting a function of the clock genes during embryonic stem cell differentiation (74, 75). Comparatively, the differentiating signals possibly driving the emergence of the clock core genes in adult stem cells are poorly understood (76). The potential role of neural signals either triggering and/or entraining clock-dependent differentiation of HSCs remains to be investigated.

Finally, cumulative evidence points toward the interplay among circadian clock system disruption, stress situations and multiple systemic diseases (77). In the hematopoietic field, unraveling the contribution of neural signals to HSC circadian regulation under acute or chronic stress might help to develop novel chronobiological therapies for hematological disorders based on neuroactive agents. As Hippocrates quoted: “Healing is a matter of time, but it is sometimes also a matter of opportunity.”

## Author Contributions

AG-G prepared the figure. AG-G and SM-F authors wrote the manuscript.

## Conflict of Interest

The authors declare that the research was conducted in the absence of any commercial or financial relationships that could be construed as a potential conflict of interest.
